# Vision With Retrodots and Factors for Declining Visual Function

**DOI:** 10.1167/iovs.63.12.17

**Published:** 2022-11-14

**Authors:** Natsuko Hatsusaka, Naoki Tanimura, Mai Yamazaki, Norihiro Mita, Yuki Ukai, Yusuke Seki, Hisanori Miyashita, Tsuyoshi Mito, Eri Kubo, Hiroshi Sasaki

**Affiliations:** 1 Department of Ophthalmology, Kanazawa Medical University, Kahoku, Ishikawa, Japan; 2 Division of Vision Research for Environmental Health, Project Research Center, Medical Research Institute, Kanazawa Medical University, Kahoku, Ishikawa, Japan; 3 Tane Memorial Eye Hospital, Osaka, Osaka, Japan

**Keywords:** retrodots, straylight, contrast visual acuity, negative spherical aberration, myopia

## Abstract

**Purpose:**

We investigated decline in the visual function of eyes with retrodots (RDs)—a subtype of cataract.

**Method:**

This study included 57 eyes with RD opacity only (mean age 72.3 ± 5.2 years) and 34 eyes with transparent lenses (mean age 71.4 ± 3.7 years). A physician diagnosed lens opacity. Using the Kanazawa Medical University Classification and Grading System, the eyes were classified into the RD-1 (37 eyes, RDs <25% of the 3-mm pupil area) and RD-2 (20 eyes, RDs ≥25% of the 3-mm pupil area) groups. Corrected distance visual acuity, contrast visual acuity, ocular refractive power, lens power, straylight, and backward light-scattering intensity and their relationship with visual function decline and optical characteristics of the eyeball were evaluated.

**Results:**

Corrected distance visual acuity was significantly lower in the RD eyes than in controls. Contrast visual acuity decreased significantly in the RD-2 eyes in all environments and in the RD-1 eyes in the evening (EVE) and EVE + glare. Straylight was significantly higher in the RD-2 eyes than in the controls and RD-1 eyes but not different between the RD-1 eyes and controls. The RD-2 eyes were significantly more myopic than the controls and RD-1 eyes.

**Conclusion:**

When the opacity of RD eyes is ≥25%, the visual acuity and contrast visual acuity decrease and straylight increases. Furthermore, myopia occurs as the refractive power of the lens increases. Moreover, visual function decline may be due to an increase in the straylight value, which is necessary for determining surgical indications.

Retrodots (RDs) were first reported by Bron and Matsuda^1^ in 1981; they are recognized as a subtype of cataract. RDs are observed as small dome-shaped lesions in the perinuclear area (anterior or posterior surface of the nucleolus) of the lens and can be easily observed as concentrically arranged fava bean–shaped shadows that measure 80 to 500 µm on retroillumination. They are composed of accumulated calcium phosphate and calcium oxalate in the lens.^2^^–^^4^ Frost et al.^5^ reported that RDs reduce visual acuity and contrast sensitivity. Klein et al.^6^ reported a high prevalence of RDs with age in the Beaver Dam Eye Study: 1.68% in people aged 43 to 53 years and 31.2% in people aged ≥75 years, suggesting that many older adults are affected by this type of opacity. The prevalence rate of RDs among older adults from the Han people in China and Taiwan was reported to be high.^7^^,^^8^ Decreased visual acuity associated with lenticular changes is influenced by increased high-order aberrations (HOAs),^9^^–^^11^ increased backward light scattering (BLS),^12^ and increased forward light scattering.^13^^,^^14^ Furthermore, increased straylight^15^^,^^16^ is a factor that decreases visual function. A previous study reported an association between increased straylight and HOAs and lower visual function in eyes with waterclefts (WCs), another subtype of cataracts.^17^ However, there have been no detailed studies on factors influencing straylight or the visual function of eyes with RDs. The fava bean–shaped granular opacities of RDs merge to form large opacities in advanced RDs such that the opaque area covers a larger proportion of the pupillary area; however, the relationship between RD progression and functional and optical characteristics of eyes with RDs remains unknown.

In this study, to compare the visual acuity, contrast acuity, and straylight of crystalline lenses of eyes with RDs and eyes without RDs, including transparent ones, and to examine the decrease in the visual function of eyes with RDs, we classified the participants into two groups by RD occupation rate in the pupillary area: <25% and ≥25%. Furthermore, we explored the relationship between optical characteristics of eyeballs with RDs, such as HOAs and BLS, and each visual function.

## Methods

**Figure 1.**
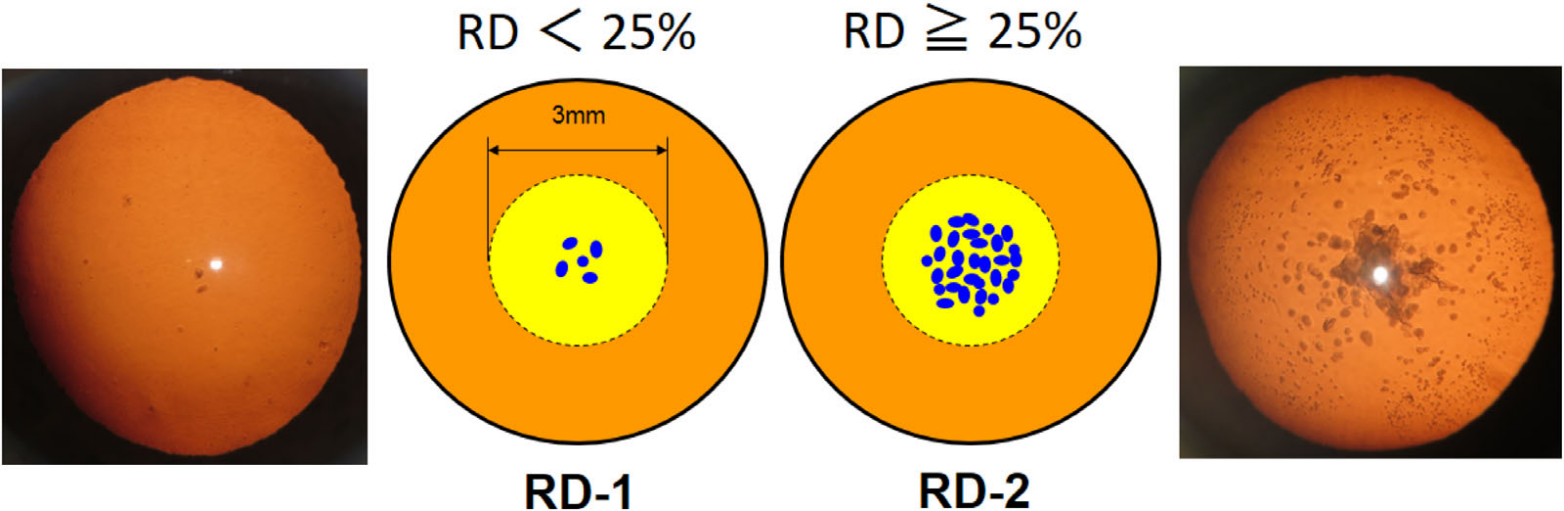
Classification of RDs. The eyes were classified based on the percentage of the opacity occupied by the RDs within the 3-mm pupillary area. RD-1: RD <25%; RD-2: RD ≥25%.

This study included patients with cataract who visited Kanazawa Medical University Hospital between October 2013 and November 2017 and participants of the longitudinal ophthalmic epidemiology Monzen Eye Study (2013–2016) in Wajima City, Ishikawa Prefecture. Thirty-four eyes with transparent lenses (mean age, 71.4 ± 3.7 years; the control group) and 57 eyes with RD opacity only (mean age, 72.3 ± 5.2 years; the RD group) were included. Lens opacity was diagnosed by the same physician using slit-lamp microscopy under maximum pupil dilation. RDs were assessed using the World Health Organization classification system^18^ and Kanazawa Medical University Cataract Classification and Grading system.^7^^,^^8^ The BLS of the fetal nucleus was measured for each participant using EAS-1000 (Nidek Co., Ltd., Aichi, Japan); participants with macroscopically apparent nuclear cataract were excluded. Patients with eye diseases other than ametropia were excluded. All the study participants reported themselves as without diabetes mellitus. Although glycated hemoglobin levels were not measured, we believe that the influence of diabetes mellitus on our results is minimal, given that no patient with diabetes mellitus retinopathy was included. EAS-1000 was used to capture retroillumination images of the eyes with RDs. The image analysis software of an EAS-1000 system was used to create a histogram for opacity. Data were converted into binary data above and below the median to calculate the area of opacity within 3 mm of the pupillary area from the extracted opacity images using the threshold method. Eyes containing RDs with an opacity of <25% in the 3-mm pupillary area were classified as RD-1 eyes (*n* = 39), whereas those with an opacity of ≥25% were classified as RD-2 eyes (*n* = 18) (Fig. 1). In the Kanazawa Medical University classification,^7^^,^^8^ RDs are classified into four groups. Grade 1 is based on the number of RDs (1–4) within the pupil area of 3 mm. Examining the visual function of eyes with RDs in this study was difficult because the effect of the number of RDs on visual function was small. Therefore, the RD eyes with an opacity of <25% were classified as RD-1. Furthermore, since we targeted patients with RD eyes only, progressive cases with >50% of opacity (grade 4 in the existing classification) were not included, and RD eyes with an opacity ≥25% were considered as RD-2. All the patients provided written informed consent. All procedures were performed according to the principles of the Declaration of Helsinki. The study was reviewed and approved by the institutional review board at Kanazawa Medical University (I375).

Corrected distance visual acuity (CDVA) was measured at 5 m using Landolt's ring chart. Contrast visual acuity (CVA) was measured using CAT-2000 (Neitz Instruments Co., Ltd., Tokyo, Japan) under photopic conditions of DAY and DAY + glare (G) and under the mesopic condition of evening (EVE) and EVE + G at 100% and 25% CVA.^19^ Spherical equivalent (SE, sphere + cylinder/2) and corneal refractive power (CP) were measured using an auto ref keratometer (ARK-730A; Nidek Co., Ltd.). Axial length (AL) was measured using IOLMaster (Carl Zeiss Meditec, Inc., Tokyo, Japan). Lens power (LP) was calculated using the equation from Olsen et al.^20^ comprising SE, CP, and AL values.

Straylight was assessed by evaluating the intraocular scatter value with C-Quant (Oculus Optikgeräte GmbH, Wetzlar, Germany) and calculating log(s). Only natural pupils were included in the measurements. Eyes with corrected refractive values beyond ±2 diopters were evaluated with SE values using trial lenses. We set the estimated standard deviation limit to 0.08,^21^ which is reported to yield reliable results with this instrument. BLS and HOAs were measured in a dark room with tropicamide 0.5% plus phenylephrine hydrochloride 0.5% to perform the test under maximum pupillary dilation, and BLS was measured by analyzing the Scheimpflug slit photography images of the lens using EAS-1000 (Nidek Co., Ltd.). BLS was calculated as the cumulative value of the cortex (anterior subcapsule to the anterior adult nucleus) and that of the nucleus (anterior fetal nucleus to the central clear zone).^22^ Furthermore, HOAs were calculated at a pupil diameter of 6 mm using KR-1W (Topcon Corporation, Tokyo, Japan). Analyses were performed for the following conditions: ocular, internal and total (ocular + internal) HOAs, coma-like aberration (S3 + S5), spherical-like aberration (S4 + S6), trefoil aberration, coma aberration, and spherical aberration.

### Analysis

Software SPSS (IBM Corp., Armonk, NY, USA) was used for the statistical analyses. ANOVA and the Tukey test were used to compare groups. Statistical significance was set at *P* < 0.05. Various relationships between RD visual acuity, straylight, and HOAs were represented with Pearson's correlation coefficient in linear regression.

## Results

There were no differences in age or sex ratio between the RD-1, RD-2, and control groups. The CDVA, ocular refractive power (spherical, cylindrical, and SE), CP, AL, and LP of the three groups are shown in Table 1. The CDVAs of the RD-1 and RD-2 eyes were significantly lower (*P* < 0.01) than that of the controls, and that of the RD-2 eyes was significantly lower than that of the RD-1 eyes (*P* < 0.01). There was no difference in astigmatism between the three groups, but the eyes with RDs became more myopic than those of the control group (*P* < 0.01). Furthermore, the LP of the RD-2 eyes was significantly higher than that of the RD-1 and controls (myopic) (*P* < 0.01).

**Table 1.** Age, Sex, Visual Acuity, Refractive Power, Astigmatism, Spherical Equivalent, Axial Length, and Lens Power of Each Group

	Group		
Parameter	RD-1	RD-2	Control
Age, mean ± SD, y	72.1 ± 4.6	72.7 ± 6.6	71.4 ± 3.7
Sex, male/female, No.	19/20	8/10	13/21
CDVA, logMAR			
Mean ± SD	0.02 ± 0.15^†^	0.13 ± 0.14^†^^,^^‡^	−0.09 ± 0.12
Range	−0.20 to 0.40	−0.20 to 0.30	−0.18 to 0.40
Sphere, D			
Mean ± SD	−0.45 ± 3.23^†^	−0.58 ± 2.30^†^	1.90 ± 1.09
Range	−13.29 to 3.50	−6.25 to 2.07	−0.25 to 3.75
Cylinder, D			
Mean ± SD	−1.21 ± 0.86	−1.12 ± 0.82	−1.29 ± 0.63
Range	−4.79 to 0.00	−3.25 to −0.25	−2.50 to −0.25
Spherical equivalent, D			
Mean ± SD	−1.05 ± 3.34^†^	−1.10 ± 2.15^†^	1.26 ± 1.12
Range	−14.35 to 3.13	−6.38 to 1.95	−1.13 to 3.38
Corneal power, D			
Mean ± SD	43.90 ± 1.71	43.73 ± 1.27	44.51 ± 1.08
Range	41.25 to 48.29	41.73 to 45.88	42.25 to 46.25
Axial length, mm			
Mean ± SD	23.91 ± 1.44^†^	23.48 ± 0.88	22.89 ± 0.65
Range	21.37 to 27.68	21.80 to 24.63	21.57 to 23.93
Lens power, D			
Mean ± SD	22.80 ± 2.28	24.21 ± 3.49^†^^,^^§^	21.77 ± 1.44
Range	18.78 to 27.88	20.25 to 32.45	19.13 to 26.10

D, diopters; CP, corneal power; AL, axial length; and LP, lens power.

*Significance test versus the control; one-way ANOVA, Tukey tests *P* < 0.05.

^†^Significance test versus the control; one-way ANOVA, Tukey tests *P* < 0.01.

^‡^Significance test versus the RD-1 group; one-way ANOVA, Tukey tests *P* < 0.01.

^§^Significance test versus the RD-1 group; one-way ANOVA, Tukey tests *P* < 0.05.

The CVA and straylight of the three groups are shown in Table 2. A 100% CVA was significantly poorer in the RD-2 eyes than in the controls under all lighting conditions (*P* < 0.01), and that of the RD-1 eyes was significantly poorer than that of the controls under EVE and EVE + G conditions (*P* < 0.05). The CVA was significantly lower in the RD-2 eyes under the DAY + G, EVE, and EVE + G conditions than in the RD-1 eyes (*P* < 0.05). At a 25% CVA, the RD-2 eyes were significantly poorer under all lighting conditions than in the controls (DAY, DAY + G, EVE [*P* < 0.01], EVE + G [*P* < 0.05]) and significantly poorer for RD-1 eyes under DAY, EVE, and EVE + G conditions (*P* < 0.01 for EVE only; *P* < 0.05 for DAY and EVE + G). The RD-2 eyes had significantly lower CVAs in the DAY and DAY + G conditions than the RD-1 eyes (*P* < 0.05). Straylight was significantly higher in both RD-1 and RD-2 eyes than in the controls (*P* < 0.01), whereas there was no difference in straylight between the RD-1 eyes and controls.

**Table 2.** Contrast Visual Acuity and Straylight of Each Group

	Group		
Parameter	RD-1	RD-2	Control
100% CVA, logMAR			
DAY			
Mean ± SD	0.20 ± 0.20	0.30 ± 0.21^*^	0.13 ± 0.19
Range	−0.1 to 0.7	0.0 to 0.7	−0.1 to 0.7
DAY + glare			
Mean ± SD	0.20 ± 0.21	0.34 ± 0.22^*^^,^^†^	0.17 ± 0.21
Range	−0.1 to 0.7	−0.1 to 0.7	−0.1 to 0.9
EVE			
Mean ± SD	0.34 ± 0.18^‡^	0.48 ± 0.22^*^^,^^†^	0.24 ± 0.15
Range	0.0 to 0.7	0.1 to 0.9	0.0 to 0.6
EVE + glare			
Mean ± SD	0.40 ± 0.21^‡^	0.54 ± 0.24^*^^,^^†^	0.28 ± 0.21
Range	0.0 to 0.9	0.0 to 0.9	−0.1 to 0.9
25% CVA, logMAR			
DAY			
Mean ± SD	0.35 ± 0.20^‡^	0.49 ± 0.20^*^^,^^†^	0.25 ± 0.16
Range	0.0 to 0.8	0.2 to 0.9	0.0 to 0.7
DAY + glare			
Mean ± SD	0.36 ± 0.23	0.52 ± 0.20^*^^,^^†^	0.29 ± 0.21
Range	0.1 to 1.0	0.2 to 0.9	−0.1 to 0.9
EVE			
Mean ± SD	0.52 ± 0.17^*^	0.59 ± 0.22^*^	0.38 ± 0.15
Range	0.2 to 0.9	0.0 to 0.9	0.1 to 0.8
EVE + glare			
Mean ± SD	0.66 ± 0.21^‡^	0.72 ± 0.27^‡^	0.52 ± 0.21
Range	0.3 to 1.0	0.0 to 1.0	0.0 to 0.9
Straylight, log(s)			
Mean ± SD	1.40 ± 0.22	1.66 ± 0.18^*^^,^^§^	1.31 ± 0.31
Range	1.00 to 1.85	1.25 to 1.93	0.59 to 2.20

* Significance test versus the control; one-way ANOVA, Tukey tests *P* < 0.01.

† Significance test versus the RD-1 group; one-way ANOVA, Tukey tests *P* < 0.05.

‡ Significance test versus the control; one-way ANOVA, Tukey tests *P* < 0.05.

§ Significance test versus the RD-1 group; one-way ANOVA, Tukey tests *P* < 0.01.

The BLS, ocular HOAs, and internal HOAs of the three groups are shown in Table 3. Cumulative values for the cortex were significantly lower in the RD-1 and RD-2 eyes than in the controls (*P* < 0.01). However, the cumulative value at the nucleus was only higher in the RD-2 eyes than in the controls (*P* < 0.01). Concerning ocular HOAs, trefoil aberration was significantly higher in the RD-2 eyes than in the controls (*P* < 0.05), and spherical-like and spherical aberrations were significantly lower (*P* < 0.05 and *P* < 0.01, respectively). Trefoil aberration was significantly higher in the RD-2 eyes (*P* < 0.05) than in the RD-1 eyes, while spherical aberration was significantly lower (*P* < 0.01). Regarding internal HOAs, the RD-2 eyes had a significantly lower spherical aberration (increased negative spherical aberration) than the controls and RD-1 eyes (*P* < 0.01). There was no significant increase in any other type of aberration.

**Table 3.** Backward Light Scattering Intensity, Ocular Higher-Order Aberration, and Internal High-Order Aberrations of Each Group

	Group		
Parameter	RD-1	RD-2	Control
BLS, cct (computer-compatible tape)			
Cumulative value from the anterior subcapsule to the adult nucleus (cortex)			
Mean ± SD	4413 ± 857^*^	3845 ± 1067^*^	5553 ± 1070
Range	2786 to 5840	2570 to 5927	3293 to 8235
Cumulative value from the fetal nucleus to the central clear zone (nucleus)			
Mean ± SD	4467 ± 907	5292 ± 2572^*^	4078 ± 885
Range	2167 to 6485	2425 to 10,278	2308 to 6259
Ocular HOA with 6-mm pupil, µm			
Total HOA			
Mean ± SD	0.59 ± 0.17	0.67 ± 0.23	0.58 ± 0.13
Range	0.21 to 0.95	0.42 to 1.02	0.31 to 0.97
Coma-like (S3 + S5)			
Mean ± SD	0.43 ± 0.18	0.56 ± 0.25^†^	0.44 ± 0.12
Range	0.08 to 0.86	0.25 to 0.85	0.20 to 0.70
Spherical-like (S4 + S6)			
Mean ± SD	0.34 ± 0.12	0.27 ± 0.10^‡^	0.37 ± 0.11
Range	0.15 to 0.61	0.17 to 0.49	0.18 to 0.71
Trefoil			
Mean ± SD	0.28 ± 0.16	0.41 ± 0.20^†^^,^^‡^	0.27 ± 0.14
Range	0.06 to 0.69	0.15 to 0.74	0.03 to 0.56
Coma			
Mean ± SD	0.30 ± 0.16	0.37 ± 0.20	0.27 ± 0.14
Range	0.06 to 0.67	0.05 to 0.74	0.06 to 0.65
Spherical			
Mean ± SD	0.28 ± 0.16	0.13 ± 0.15^*^^,^^§^	0.30 ± 0.12
Range	−0.12 to 0.53	−0.14 to 0.33	0.00 to 0.56
Internal HOA with 6-mm pupil, µm			
Total HOA			
Mean ± SD	0.32 ± 0.10	0.42 ± 0.13^†^	0.37 ± 0.17
Range	0.16 to 0.57	0.26 to 0.69	0.16 to 0.88
Coma-like (S3 + S5)			
Mean ± SD	0.23 ± 0.10^*^	0.28 ± 0.13	0.31 ± 0.14
Range	0.06 to 0.46	0.13 to 0.56	0.10 to 0.72
Spherical-like (S4 + S6)			
Mean ± SD	0.16 ± 0.08	0.23 ± 0.10	0.20 ± 0.11
Range	0.06 to 0.38	0.15 to 0.41	0.09 to 0.65
Trefoil			
Mean ± SD	0.13 ± 0.06	0.11 ± 0.06	0.15 ± 0.10
Range	0.02 to 0.35	0.06 to 0.19	0.02 to 0.50
Coma			
Mean ± SD	0.18 ± 0.10	0.24 ± 0.16	0.23 ± 0.13
Range	0.02 to 0.44	0.04 to 0.56	0.05 to 0.67
Spherical			
Mean ± SD	−0.01 ± 0.12	−0.13 ± 0.15^*^^,^^§^	0.00 ± 0.09
Range	−0.37 to 0.23	−0.37 to 0.13	−0.26 to 0.15

* Significance test versus the control; one-way ANOVA, Tukey tests *P* < 0.01.

† Significance test versus the RD-1 group; one-way ANOVA, Tukey tests *P* < 0.05.

‡ Significance test versus the control; one-way ANOVA, Tukey tests *P* < 0.05.

§ Significance test versus the RD-1 group; one-way ANOVA, Tukey tests *P* < 0.01.

**Figure 2.**
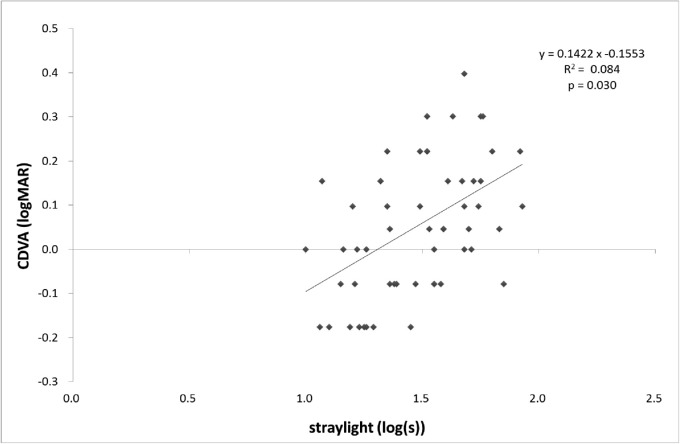
Correlation between straylight and CDVA in eyes with RDs. Pearson's correlation coefficient in linear regression.

**Figure 3.**
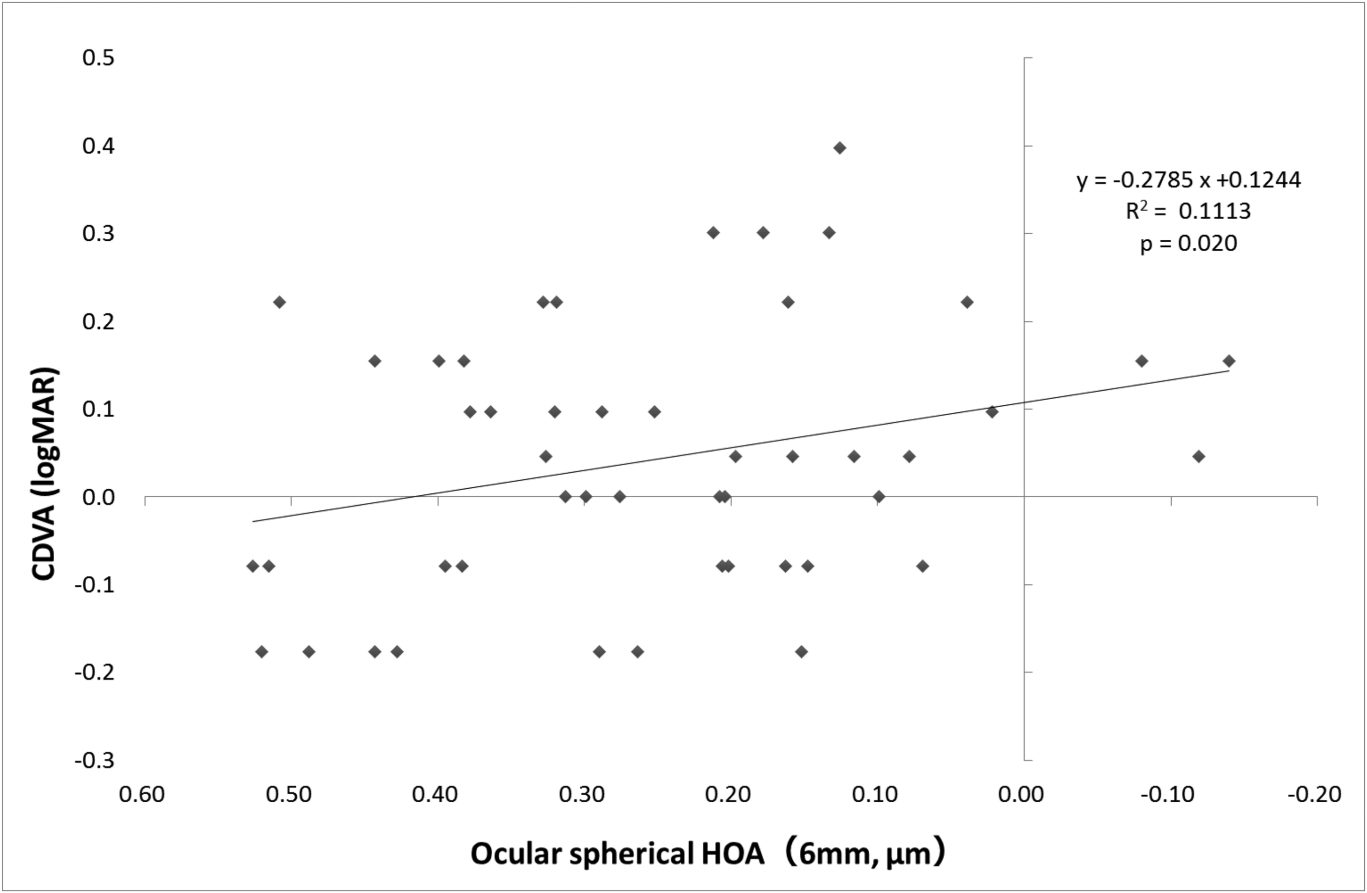
Correlation between ocular spherical HOAs and CDVA in eyes with RDs. Pearson's correlation coefficient in linear regression.

**Figure 4.**
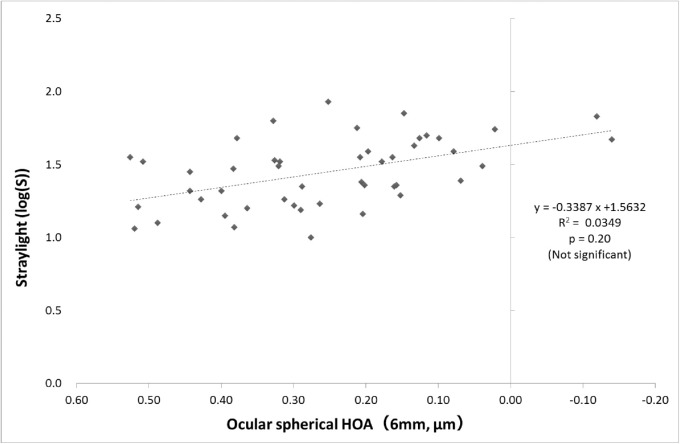
Correlation between ocular spherical HOAs and straylight in eyes with RDs. Pearson's correlation coefficient in linear regression.

Correlations between CDVA, straylight, negative spherical aberration (both ocular and internal increased significantly in RD eyes), and BLS of the nucleus were investigated in the RD eyes. As shown in Figure 2, CDVA and straylight correlated significantly, and CDVA decreased significantly with increased straylight (*P* < 0.05). Furthermore, a significant correlation was observed between CDVA and spherical aberration, as shown in Figure 3, where CDVA decreased significantly with increased negative spherical aberration (*P* < 0.05). However, no significant correlation was observed between the increase in negative spherical aberration and straylight, suggesting that an increased straylight was unrelated to spherical aberrations (Fig. 4). Moreover, no correlation was observed between BLS of the nucleus and CDVA or straylight.

**Figure 5.**
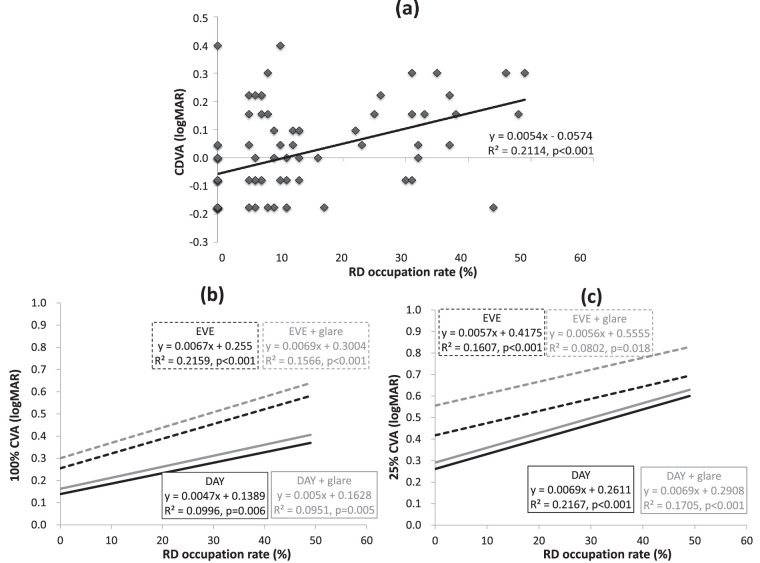
Correlations between RD occupation rate, CDVA, 100% CVA, and 25% CVA in all the participants. (**a**) Correlation between RD occupation rate and CDVA. (**b**) Correlation between RD occupation rate and 100% CVA (DAY, DAY + glare, EVE, EVE + glare). (**c**) Correlation between RD occupation rate and 25% CVA.

**Figure 6.**
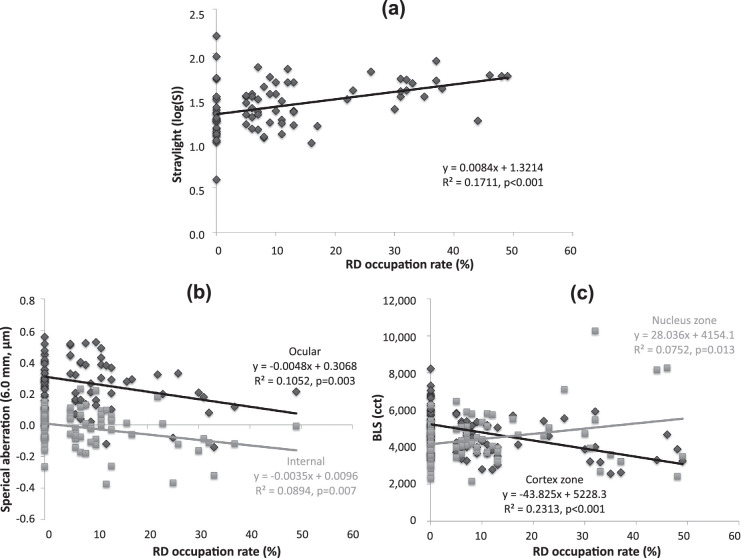
Correlations between RD occupation rate, straylight, spherical aberration, and BLS in all the participants. (**a**) Correlation between RD occupation rate and straylight. (**b**) Correlation between RD occupation rate and spherical aberration (ocular and internal). (**c**) Correlation between RD occupation rate and BLS (the cortex zone and nucleus zone).

To investigate the effect of RDs on visual function in more detail, we performed a simple linear regression analysis to investigate the relationship between RD occupation rate and CDVA, CVA, straylight, spherical aberration, and BLS within the pupil area of 3 mm in all the participants, including clear eyes. The results are shown in Figures 5 and Figure 6. As the RD occupation rate increased, CDVA and CVA (total vision environment: DAY, DAY + G, EVE, EVE + G) decreased significantly (Fig. 5). Furthermore, straylight increased significantly (Fig. 6), demonstrating that an increase in RD occupation rate affects the decline in visual function. In addition, negative spherical aberration in the ocular and intraocular area also increased significantly, and there was a significant correlation with BLS (Fig. 6). Regarding BLS, the nucleus zone (the fetal nucleus to the central clear zone) increased significantly, while the cortex zone (the anterior subcapsule to the adult nucleus) showed a significant decrease.

## Discussion

This study evaluated the visual function of eyes with RDs, a subtype of cataract, using CDVA, CVA, and straylight by the area of the RDs, and investigated optical characteristics, such as LP, HOAs, and BLS severity. The analysis revealed that CDVA and CVA decreased with the area of the RD eye, and visual function decreased with increased straylight. Furthermore, this is the first study to confirm that LP increased significantly in eyes with RDs.

Nuclear cataract, the most common type of cataract, is known to affect myopia^23^; however, the relationship between eyes with RDs and refractive power is unclear. Vrensen et al.^2^^,^^3^ reported that RDs result from an accumulation of calcium phosphate and calcium oxalate and that their refractive indices are approximately 1.63 and 1.5, respectively; thus, their greater refractive indices than that of the lens could be a reason for the myopic shift. However, in RD-1 eyes, LP was not significantly more myopic than the transparent eyes, suggesting that an aggregation of a certain amount of RDs at the central part of the pupillary region may increase the refractive index in the perinuclear area of the lens. Moreover, an increase in RDs may increase the area of high refractive indices in the central pupil, which is expected to worsen myopia even further.

An increase in RDs was associated with a significantly lower CDVA and CVA and with significantly more straylight. Straylight is associated with decreased quality of vision in eyes with cataracts.^15^^,^^16^ CDVA and straylight are weakly correlated to each other in eyes with cataract.^24^ Thus, increased straylight may lead to more scattering in the retinal direction, thereby lowering contrast in the retinal image and causing a significantly lower CVA or CDVA. We set a value of 1.4 as a normal level of straylight and examined the rate of patients with a straylight value over 1.4, placing them in a good visual acuity group (logMAR <0.1). As a result, 71% of the RD-2 eyes and 35% of the RD-1 eyes with a good visual acuity (<0.1 logMAR) had increased straylight. Among the RD eyes without decreased visual acuity, visual function deteriorated due to increased straylight in many patients. When we previously studied straylight in eyes with WCs, we observed that some patients with a good visual acuity experienced a decrease in visual function due to increased straylight by WC localization at the center of the pupillary zone.^17^ These results were consistent with ours; therefore, we propose that indications for surgery should be determined by considering increased straylight for subtypes of cataracts without prominent opacities.

A significant increase in the severity of BLS at the nucleus and a significant increase in HOAs (negative spherical aberration) were observed with an increase in RDs in this study. An increase in backward scatter lowers retinal illuminance, thereby leading to the deterioration in visual function. Increased backward scatter may cause deteriorated visual function in nuclear cataracts, cortical cataracts with intense opacity in the pupillary region, and posterior subcapsular cataracts. Backward scatter also increased with an increase in the RD area of the eyes with RDs. However, the lack of correlation with CDVA or straylight confirmed that it was not severe enough to play a role in deteriorating visual function. A significant increase in negative spherical aberrations was also observed at a pupil diameter of 6.0 mm. This is like nuclear cataracts, which cause myopia. Especially in long-axis eyes with nuclear cataracts, negative spherical aberrations and severe myopic changes were observed.^25^ RD aggregation in the anterior and posterior surrounding lens nucleus was believed to have increased the refractive index, thereby causing the increase in negative spherical aberrations in the eyes with RDs in the present study.

The analysis of the relationships between CDVA, straylight, negative spherical aberrations, and BLS of the nucleus in the eyes with RDs suggested that increased straylight and negative spherical aberrations were components of the lower CDVA in the eyes with RDs. A previous study reported a mild correlation between CDVA and straylight in the eyes with cataract^24^; this was same for eyes with RDs. Furthermore, several studies have reported that increased HOAs worsen distortion of retinal images and deteriorate visual acuity.^26^ Therefore, increased RDs in the pupillary region may be linked to higher negative spherical aberrations, thereby causing deteriorated visual acuity in some cases. Furthermore, increased negative spherical aberrations were not significantly correlated with straylight. The results of the present study suggest that the main factor of deteriorated visual acuity in the RD eye is increased straylight, and it is caused by an increased forward scatter. Kamiya et al.^27^ reported a significantly higher forward scatter in cataracts (Optical Quality Analysis System [OQAS]; HD Analyzer II, Visiometrics SL, Terrassa, Spain).^28^ Previously, we used OQAS to confirm the significant increase in forward scatter in eyes with RD opacity and observed that for in-depth investigation, further studies with criteria related to forward scatter are necessary.

From this result, a significant correlation was shown between the increase in RD occupation rate and CDVA, CVA, and straylight, clarifying that an increase in RDs within the pupil area of 3 mm affects visual function. As the RD occurrence rate increases, the straylight increases and the CVA and CDVA decrease. Furthermore, the increase of negative spherical aberration also leads to myopia. There was no significant correlation between spherical aberration of the eyeball and straylight in the eyes with RDs only. However, there was a significant correlation when examined in all the participants, including those with transparent eyes (Pearson's correlation coefficient, –0.296; *P* = 0.007). It was reconfirmed that the increase in negative spherical aberration increased the straylight, leading to a decrease in visual function. On the other hand, in BLS, the increase in RDs caused an increase in the scattering in the nucleus and conversely decreased the scattering in the cortex. A comparison of the BLS mean values ​​of RD-1 and RD-2 showed no significant difference between the two groups but a significant correlation. It is also possible that an increase in the RD opacity area increases the difference in the BLS in the cortex to the nucleus, leading to a decrease in visual function. However, the relationship between the increase in RDs and BLS needs to be examined in detail in the future.

This study had several limitations. First, eyes with RDs are often complicated with nuclear opacity; thus, some of the eyes with RDs in this study may have had a slight nuclear opacity. Therefore, the high backward scatter in the nuclei of the eyes with RDs in this study may be linked to the fact that some of the eyes might have been affected by nuclear cataracts with milder scattering. However, the participants of this study did not include patients with severe myopia, which is prone to worsen due to opacities in the nucleus, and we believe that the increase in the intensity of BLS in the nucleus has a small effect on the refractive power of the lens. There are several types of RDs: large fava bean–shaped RDs, fine dots, and fine RDs that aggregate to form large RDs. These RD subtypes are expected to have different effects on visual function characteristics, such as forward scatter, HOAs, and LP, but such factors were not considered in the present analysis. Finally, the relationship with forward scatter, which was proposed to cause increased straylight in the eyes with RDs, should be further investigated.
